# Glucocorticoid‐Induced Autophagy Protects Osteocytes Against Oxidative Stress Through Activation of MAPK/ERK Signaling

**DOI:** 10.1002/jbm4.10077

**Published:** 2018-12-11

**Authors:** Rekha Kar, Manuel A. Riquelme, Rui Hua, Jean X. Jiang

**Affiliations:** ^1^ Department of Biochemistry and Structural Biology University of Texas Health Science Center San Antonio TX USA

**Keywords:** OSTEOCYTE, AUTOPHAGY, APOPTOSIS, MAPK/ERK, CELL PROTECTION

## Abstract

Autophagy confers protective or detrimental effects on cells depending on the cellular context. We showed here that oxidative stress‐induced cell death in osteocytic MLO‐Y4 cells coincided with decreased autophagy. Decreased autophagy was also observed in osteocytes of superoxide dismutase 1‐ (SOD1‐) deficient mice. Oxidative stress‐induced osteocyte death was exacerbated by an autophagy inhibitor, chloroquine, suggesting a protective function of basal autophagy levels against oxidative stress‐induced cell death. Pretreatment with dexamethasone reduced the susceptibility of osteocytes to oxidative stress‐induced cell death and conferred protection against TNFα/cycloheximide‐induced cell death. Inhibition of MAPK/ERK attenuated the formation of autophagosome, leading to increased osteocyte cell death. Taken together, our results suggest that autophagy, induced by moderate levels of glucocorticoids, leads to the preconditioning of osteocytes and conveys a novel cell‐protective function against cell death induced by oxidative stress and other insults. © 2018 The Authors. *JBMR Plus* is published by Wiley Periodicals, Inc. on behalf of the American Society for Bone and Mineral Research.

## Introduction

Autophagy, a conserved cellular process, acts in concert with the ubiquitin–proteasome system to degrade long‐lived unfolded proteins and damaged organelles.^1^ Development of autophagy requires the autophagy‐related genes (ATG) and microtubule‐associated light chain 3 (LC3). Impaired autophagy results in several neurodegenerative diseases including Alzheimer's disease and Parkinson's disease.^2^ Autophagy has been described as a double‐edged sword^3^, ^4^, ^5^; inadequate autophagy leads to the death of long‐lived postmitotic cells, whereas excess autophagy causes the destruction of cellular components and eventual cell death.

Treatment with glucocorticoids induces autophagy in primary osteocytes, osteocytic MLO‐Y4 cells, and osteocytes in situ.^6^, ^7^ Moreover, in vivo studies suggest that a lower dose of glucocorticoid activates autophagy and a higher dose increases apoptosis.^8^ Recently, it was shown that conditional deletion of an autophagy gene, ATG7, in osteocytes resulted in decreased bone mass, a characteristic exhibited in mice mimicking skeletal aging.^9^ However, the mechanistic role of autophagy in osteocytes remains largely elusive.

Osteocyte cell death increases progressively with age in mice,^10^ and oxidative stress during aging is a major mechanism contributing to the death of bone cells (reviewed by^11^, ^12^). We reported earlier that oxidative stress directly induces cell death in osteocytes.^13^ We showed here that oxidative stress reduced basal autophagy levels in both cultured osteocytes and in bone tissues of SOD1 knockout mice. Inhibition of autophagy exacerbates oxidant‐induced death of osteocytes. Conversely, activation of autophagy by pretreatment with moderate glucocorticoid levels offers protection against oxidants. These data suggest a protective function of autophagy. Furthermore, glucocorticoids activated the MAPK/ERK signaling pathway, and increased regulated autophagy and osteocyte survival under oxidative stress.

## Materials and Methods

### Materials

MLO‐Y4 cells were provided by Dr. Lynda Bonewald, University of Missouri–Kansas City. Chemiluminescent substrates were purchased from GE Healthcare (Fairfield, CT, USA). Apoalert annexin/propidium iodide (PI) detection kit was from Clontech (Mountain View, CA, USA). Antibody for LC3 (catalog# 2775), ATG7 (catalog# 2631), phospho‐p44/42 MAPK (catalog# 9101), and p44/42 MAPK (Erk1/2; catalog# 9102) were from Cell Signaling (Danvers, MA, USA), antibody for Shc/p66 (pSer^36^; catalog# 566807) was from EMD Millipore‐Calbiochem (Darmstadt, Germany), antibodies for GAPDH (catalog# AM4300) and for β‐actin (catalog# MA5‐15739) were from ThermoFisher Scientific‐Ambion (Waltham, MA, USA). Chloroquine (C6628), dexamethasone (Dex; D2915), menadione (M5625), and acridine orange solution were from Sigma‐Aldrich (St. Louis, MO, USA; catalog# A9231). U0126 (U6770) was from LC Laboratories (Woburn, MA, USA) and caspase‐3 antibody was from Santa Cruz Biotechnology (Santa Cruz, CA, USA). Rotenone was from Calbiochem (557368). All other reagents were obtained either from Sigma‐Aldrich or Fisher Scientific with the highest grade available. Stock solutions of these different chemicals including Dex, chloroquine, menadione, rotenone, and U0126 were made based on the manufacturer's instructions. Briefly, Dex was prepared as follows: We added 1 mL absolute ethanol per mg product and gently swirled to dissolve. We then added 49 mL sterile PBS per mL of ethanol, while mixing, to achieve a final concentration of 20 µg/mL. Chloroquine and menadione both were solubilized in PBS. Rotenone and U0126 were both dissolved in DMSO in a 1000X stock solution. All the vehicle controls used the identical solution as treated groups.

### Cell culture

MLO‐Y4 cells were seeded at 1 × 10^4^/cm^2^ on collagen coated (rat tail collagen type I, 0.15 mg/mL) 35 mm or 60 mm cell culture dishes from Corning Inc. (Corning, NY, USA; catalog# 430165 or 430166, respectively) and were grown in α‐MEM (Thermo Scientific, Rockford, IL, USA) supplemented with 2.5% FBS and 2.5% BCS (both serums were from Hyclone GE Healthcare, Chicago, IL, USA), 2mM L‐glutamine, penicillin 50 U/mL, and 50 μg/mL streptomycin (all three were purchased from Thermo Scientific) and were incubated in a 5% CO_2_ incubator at 37°C, as described previously.^14^

### SOD1 mice and isolation of bone extracts

This SOD1 deficient mouse model is a global knockout.^15^ These mice under C57BL/J6 background were generously provided by Dr. Brian Herman, University of Texas Health Science Center–San Antonio (UTHSCSA). The mice were housed in a temperature‐controlled room with a light/dark cycle of 12 hours at the UTHSCSA Institutional Lab Animal Research (LAR) facility under specific pathogen free conditions. Food and water were provided *ad libitum*. All animal protocols were performed in accordance with the National Institutes of Health guidelines for the care and use of laboratory animals, and approved by the UTHSCSA Institutional Animal Care and Use Committee. The tibial bone tissues of 7 month old male mice were isolated free of soft tissues, and bone marrow was removed by flushing with PBS. The bone samples were then pulverized using a frozen mortar and pestle in liquid nitrogen. Bone samples were homogenized with 0.25 mL cold lysis buffer (5mM EGTA, 5mM EDTA, and 5mM Tris pH 8) in the presence of phosphatase and protease inhibitors (10mM NaF, 10mM Na_3_VO_4_, 2mM Na_4_P_2_O_7_, 2mM Na_2_ β‐glycerophosphate, 2mM phenylmethylsulfonyl fluoride, 2mM leupeptin, 2mM N‐ethylmaleimide). The homogenates were centrifuged at 16,000*g* for 10 min at 4°C, and the pellet was discarded. Protein concentrations were determined by the Micro BCA Protein Kit (Thermo Scientific) and the lysates were used for Western blotting analysis.

### Western blotting

Treated MLO‐Y4 cells were collected in lysis buffer as described above in the presence of phosphatase and protease inhibitors. For each sample, 30 μg of total protein were resolved by SDS–polyacrylamide gel electrophoresis (SDS–PAGE) under reducing conditions and then electroblotted onto nitrocellulose membranes. Membranes were blocked in 10% milk, incubated with primary antibodies in 5% BSA overnight at 4°C, washed, and then incubated with peroxidase‐conjugated suitable secondary antibodies in 10% milk for 1 hour at room temperature. A chemiluminescent substrate, SuperSignal West Femto Maximum Sensitivity Substrate from Thermo Scientific (catalog# 34095) was used to detect antigen–antibody complexes on the membrane. Densitometry for the imaged blots was performed using LI‐COR Imaging Systems with LI‐COR Image Studio software (Lincoln, NE, USA).

#### Annexin V‐FITC and PI double staining, acridine orange staining, and FACS analysis

MLO‐Y4 cells cultured in phenol red‐free media were pretreated with or without 1μM Dex for 16 hours or 50μM chloroquine for 1 hour, and then were exposed to various doses of H_2_O_2_ (0.1mM to 0.5mM) with or without 50μM U0126, 20μM menadione, or 20μM rotenone. MLO‐Y4 cells were trypsinized, stained with annexin V‐FITC and PI using the Apoalert Annexin V Apoptosis Kit (Clontech) and were subjected to FACS analysis by flow cytometry (FacsCalibur Analyzer; BD Biosciences, Franklin Lakes, NJ, USA). MLO‐Y4 cells were incubated with 1μg/mL acridine orange (Sigma‐Aldrich, catalog# A9231) for 15 min at 37°C, then were washed with PBS, trypsinized, and subjected to FACS analysis. The data were analyzed by BD CellQuest software (San Jose, CA, USA).

### Statistical analysis

All the data were analyzed using GraphPad Prism 5.04 software (GraphPad Software, La Jolla, CA, USA). One‐way ANOVA and Student‐Newman Keul's test were used for more than two compared groups and paired Student's *t* test was used for comparison between two groups. Unless otherwise specified in the figure legends, the data are presented as the mean ± SEM of three independent experiments. Asterisks indicate the degree of significant differences: *p < 0.05; **p < 0.01; ***p < 0.001.

## Results

### Autophagy protects osteocytes against cell death induced by oxidative stress and other insults

**Figure 1:**
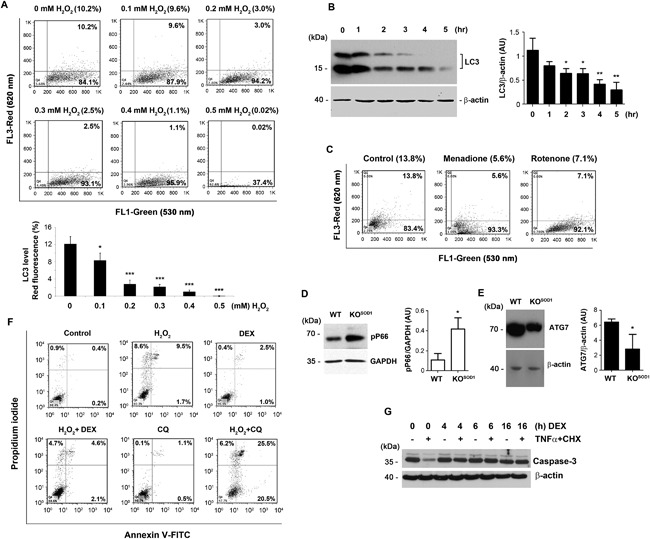
Dexamethasone primes osteocytes against cell death induced by oxidative stress and other stress‐induced cell death. (*A*) MLO‐Y4 cells were treated with various concentrations of H_2_O_2_ for 5 hours, stained with acridine orange (1 μg/mL) and subjected to flow cytometry analyses. Graph shows percentage of stained cells (lower panel): Control versus 0.1mM H_2_O_2_, *p < 0.05; control versus 0.2, 0.3, 0.4, and 0.5mM H_2_O_2_, ***p < 0.001. Data are presented as mean ± SEM, *n* = 3. (*B*) MLO‐Y4 cells were treated with 0.4m*M* H_2_O_2_ for 0 to 5 hours. Cell lysate was analyzed by Western blots using anti‐LC3 or anti‐β‐actin antibody. The intensity of the bands was quantified (right panel): Control versus 2 and 3 hours, *p < 0.05; control versus 4 and 5 hours, **p < 0.001. Data are presented as mean ± SEM, *n* = 3. (*C*) MLO‐Y4 cells were treated with menadione or rotenone for 7 hours. Cells were stained with acridine orange and then subjected to flow cytometry analyses. Bone extracts from long bones of wild‐type (WT) and SOD1 knockout (KO^SOD1^) mice were analyzed by immunoblotting using anti‐phospho‐P66 antibody or anti‐GAPDH antibody (*D*) or anti‐ATG7 or anti β‐actin antibody (*E*). The band intensity was quantified (right panels): WT versus KO^SOD1^, *p < 0.05. The data are presented as mean ± SEM, *n* = 3. (*F*) MLO‐Y4 cells were pretreated with an autophagy inhibitor, chloroquine (CQ), or dexamethasone (DEX) and were then treated with or without 0.5mM of H_2_O_2_ for 5 hours. Cells were labeled with annexin V‐FITC and propidium iodide, and were analyzed using flow cytometry analyses. (*G*) MLO‐Y4 cells were pretreated with dexamethasone (DEX) for various periods and were then treated with TNFα (10 ng/mL) and CHX (10 μg/mL) (CHX) for 16 hours. Cell lysates were immunoblotted with anticaspase 3 or anti β actin antibody.

Treatment with H_2_O_2_ decreased basal autophagic activity as revealed by the decreased formation of autophagic vesicular organelles measured by acridine orange red fluorescence in a dose dependent manner (Fig. 1 *A*). As compared to the control with acridine orange red fluorescence (10.2%), treatment with 0.2mM H_2_O_2_ decreased the fluorescence intensity to 3% and eventually decreased to 0.02% with 0.5mM of the oxidant. H_2_O_2_ also decreased expression of LC3, a cellular marker of autophagy. MLO‐Y4 cells were treated with 0.4mM H_2_O_2_ for up to 5 hours and a time dependent decrease in LC3 expression was observed (Fig. 1 *B*). This decrease in autophagy was also evident in osteocytes treated with two other types of oxidants: menadione, a redox cycling quinine, and rotenone, a mitochondrial complex I inhibitor (Fig. 1 *C*). Deletion of SOD1 in a knockout mouse model increases ROS and oxidative stress.^16^ A significant increase in the expression of phospho‐P66, an oxidative stress marker, and decreased expression of ATG7, a cellular marker for autophagy, was observed in long bone extracts of SOD1 knockout mice (Fig. 1 *D*, *E*). These results suggest that oxidative stress has a negative impact on autophagy in osteocytes.

To elucidate the role of autophagy during oxidant‐induced osteocyte death, we either inhibited autophagy by chloroquine or activated autophagy using Dex.^6^, ^7^ Preincubation with chloroquine (CQ) further exacerbated H_2_O_2_ induced death, with an increase of PI annexin V‐FITC double positive cells from 9.5% to 25.5%; chloroquine by itself did not exert much cytotoxicity (Fig. 1 *F*). However, cell death induced by H_2_O_2_ decreased (from 9.5% to 4.6%) with Dex pretreatment. To further confirm the role of autophagy in protecting osteocytes against cell death, MLO‐Y4 cells were pretreated with Dex for different periods and were then treated with TNFα and cycloheximide (CHX) as the cotreatment induces apoptosis of osteocytes.^6^ The pretreatment with Dex decreased caspase‐3 cleavage (Fig. 1 *G*), suggesting the reduction of apoptotic cell death. These results show that autophagy protected cells not only from oxidant induced death, but also from other stresses.

### MAPK/ERK pathway promotes autophagy and protects osteocytes against oxidative stress

**Figure 2:**
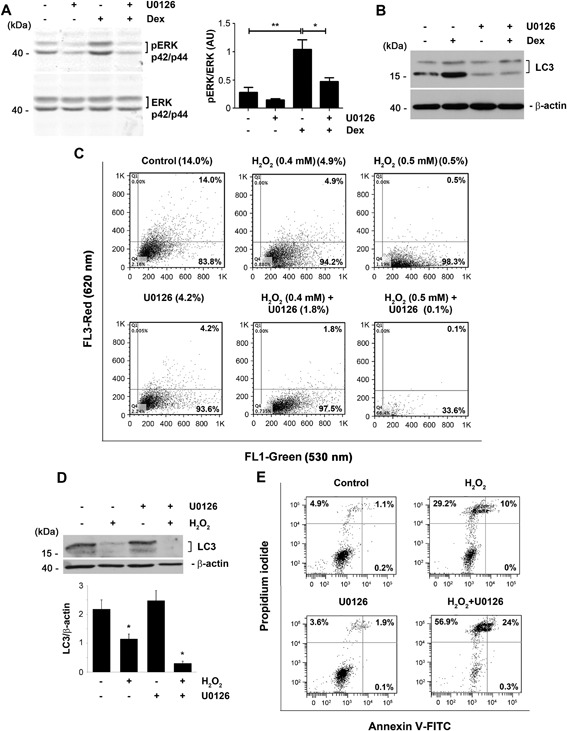
Inhibition of MAPK/ERK signaling attenuates increased autophagy by dexamethasone, and augments the reduction of autophagy and the increase of cell death by H_2_O_2_. MLO‐Y4 cells were pretreated with MAPK/ERK pathway inhibitor, U0126 for 1 hour and then treated with or without dexamethasone (DEX) for 4 hours. Cell lysates were immunoblotted with anti phospho‐ERK p42/p44 or anti total ERK p42/p44 antibody (*A*) or anti LC3, or anti β‐actin antibody (*B*). The density of the bands was quantified (*A*, right panel): *p < 0.05; **p < 0.01. *n* = 3. (*C*) MLO‐Y4 cells were incubated with U0126 for 1 hour before treatment with H_2_O_2_ for 5 hours. Cells were stained with acridine orange and analyzed by flow cytometry. (*D*,*E*) MLO‐Y4 cells were incubated with U0126 for 1 hour before treatment with H_2_O_2_ for 5 hours. (*D*) Cell lysate was collected after the treatment and was subjected to immunoblotting using anti LC3 or anti β‐actin antibody. Control versus H_2_O_2_, *p < 0.05; H_2_O_2_ versus U0126 + H_2_O_2_, *p < 0.05. The data are presented as mean ± SEM, *n* = 3. (*E*) Cells were stained with annexin V‐FITC and PI and were analyzed by flow cytometry.

MAPK/ERK signaling is involved in autophagosome maturation.^17^, ^18^ Dex significantly activated ERK signaling in osteocytes, as shown by phosphorylated ERK (pERK) p42/p44 levels; this activation was attenuated by U0126, a specific inhibitor of the ERK pathway (Fig. 2 *A*). Incubation with U0126 totally abolished the increase in LC3 levels induced by Dex in osteocytes (Fig. 2 *B*). Moreover, FACS analyses showed that U0126 reduced basal autophagy levels, indicated by acridine orange red fluorescence, from 14% in control to 4.2% with U0126 (Fig. 2 *C*). Furthermore, the H_2_O_2_‐mediated decrease of LC3 was further augmented by inhibition of ERK signaling by U0126 (Fig. 2 *D*). Consistent with the protective role of autophagy, inhibition of the MAPK/ERK signaling exacerbated H_2_O_2_ induced cell death as shown by increased cells double labeled with propidium iodide and annexin‐V‐FITC (Fig. 2 *E*). These data suggest that MAPK/ERK signaling positively regulates autophagy and protects osteocytes via elevated autophagy.

## Discussion

We report that increased oxidative stress results in decreased basal autophagic activity in osteocytes and activation of autophagy confers protection against oxidative stress and other cytotoxic reagents. Inhibition of autophagy by specific inhibitors that block the MAPK/ERK pathway increased oxidant induced osteocyte cell death. Moreover, the reduction of autophagy in the long bones of SOD1 knockout mice correlated with increased oxidative stress. Our results clearly show that oxidative stress has a negative impact on autophagy and osteocyte viability. Interestingly, conditional ATG7 knockout mice exhibited increased oxidative stress, decreased cortical bone thickness and cancellous bone volume, and increased cortical porosity.^9^ Coincidentally, a recent study reported that osteocyte autophagy is inversely correlated with oxidative stress and bone loss in ovariectomized rats.^19^ Moreover, other groups have reported an inverse correlation between autophagy and oxidative stress in osteoblasts and osteoblastic lineage cells.^20^, ^21^ Thus, it appears that levels of both oxidative stress and autophagy are intricately controlled in osteocytes; increasing oxidative stress is associated with decreasing autophagy and vice versa.

In the current study, we primarily used osteocytic MLO‐Y4 cells. This cell model has been commonly used to study osteocytes^22^ because of known technical difficulties in generating sufficient numbers of pure primary osteocytes for experiments such as the FACS analysis and Western blotting performed in this study. Minimal apoptosis was detected at the dosage of Dex we used. This observation is consistent with a previously reported study in vivo, showing that lower doses of Dex (ie, 1.4 mg/kg) do not increase osteocyte apoptosis, in contrast with higher doses, and this is inversely correlated with levels of autophagy.^8^ Here, we observed that lower doses of Dex elevated autophagy that preconditioned osteocytes. This preconditioning increased the resistance of osteocytes against the death induced by oxidants and other cytotoxic reagents. We found here that when we pretreated osteocytes with Dex, osteocytes become more resistant to death in response to oxidative stress or other apoptosis‐inducing reagents. This protective role is likely to be mediated by the enhancement of antioxidant mechanisms based on elevated autophagy. Indeed, an in vivo study showed that low dosage exposure to Dex increases autophagy in cortical mouse bone, and this increase is well correlated with antioxidant gene expression.^8^ In contrast to our observation in osteocytes, impairment of autophagy via ATG5 knockdown instead decreases basal as well as drug‐induced oxidative stress in osteosarcoma cells.^23^ As discussed above, autophagy possesses a biphasic role: Either total impairment or overenhancement of autophagy may exert adverse effects on cells.

We showed that the MAPK/ERK pathway is a positive regulator of autophagy. Inhibiting this pathway abolished autophagy under basal conditions and also its induction in response to Dex. This study suggests that the autophagy caused by Dex depends on MAPK/ERK signaling. The involvement of MAPK/ERK signaling in regulating autophagy has been reported in other cell types.^17^, ^18^, ^24^ A study reports that estradiol inhibits osteoblast apoptosis through the ER‐ERK‐mTOR pathway and the activation of this pathway enhances autophagy.^24^ It is likely that the activation of MAPK/ERK pathway results in the initiation of autophagy, and this pathway plays a major role in defining osteocyte cell fate concerning survival or death. Together, our study elucidates a major protective mechanism of osteocytes against oxidative stress and other insults that is mediated through autophagy, MAPK/ERK, and preconditioning mechanisms.

## Disclosure

The authors declare no competing or financial interests.
